# A Polymer Optical Fiber Temperature Sensor Based on Material Features

**DOI:** 10.3390/s18010301

**Published:** 2018-01-19

**Authors:** Arnaldo Leal-Junior, Anselmo Frizera-Neto, Carlos Marques, Maria José Pontes

**Affiliations:** 1 Graduate Program of Electrical Engineering, Federal University of Espirito Santo, Vitória-ES 29075-910, Brazil; frizera@ieee.org (A.F.-N.); mjpontes@ele.ufes.br (M.J.P.); 2 Instituto de Telecomunicações and Physics Department & I3N, University of Aveiro, 3810-193 Aveiro, Portugal; carlos.marques@ua.pt

**Keywords:** polymer optical fiber, temperature sensor, dynamic mechanical analysis

## Abstract

This paper presents a polymer optical fiber (POF)-based temperature sensor. The operation principle of the sensor is the variation in the POF mechanical properties with the temperature variation. Such mechanical property variation leads to a variation in the POF output power when a constant stress is applied to the fiber due to the stress-optical effect. The fiber mechanical properties are characterized through a dynamic mechanical analysis, and the output power variation with different temperatures is measured. The stress is applied to the fiber by means of a 180° curvature, and supports are positioned on the fiber to inhibit the variation in its curvature with the temperature variation. Results show that the sensor proposed has a sensitivity of 1.04 × 10^−3^ °C^−1^, a linearity of 0.994, and a root mean squared error of 1.48 °C, which indicates a relative error of below 2%, which is lower than the ones obtained for intensity-variation-based temperature sensors. Furthermore, the sensor is able to operate at temperatures up to 110 °C, which is higher than the ones obtained for similar POF sensors in the literature.

## 1. Introduction

Temperature assessment plays an important role in industrial applications, such as automotives, air conditioning control, chemical processes, and food storage [1]. Furthermore, temperature sensors are employed in a wide range of medical applications [2]. Traditional temperature sensors such as thermocouples, thermistors, and resistance temperature detectors are common [3]. However, these sensors may present errors in applications that involve high electromagnetic disturbances and safety issues when applied in harsh environments [3].

In order to overcome the limitations of conventional technologies for temperature measurement, different optical fiber temperature sensors have been proposed. The different approaches for temperature sensors include interferometric configurations, such as Fabry–Perot [4], Mach–Zehnder [5], fiber Bragg gratings (FBGs) [6], intensity-variation-based sensors [1], and nonlinear effects [7]. Since such sensors are based on optical signals instead of electrical signals, they are compact, lightweight, and have multiplexing capabilities, electromagnetic immunity, and intrinsic safety [8].

Although there are several optical fiber-based techniques for measuring temperature, the techniques related to FBGs, interferometers, and nonlinear effects generally imply more complex signal processing, implementation, and the cost of interrogation equipment can make these technologies unsuitable for low-cost applications [3]. For a low-cost system of temperature measurement with simplicity in signal processing and easy implementation, intensity-variation-based sensors are preferred [9]. Although such sensors may present errors due to variations in light source power [9], this problem can be overcome with techniques based on self-referencing schemes [10], dual-wavelength compensation [3,11], or light reflection [12].

Higher flexibility, fracture toughness, and strain limits enable the application of higher curvatures on polymer optical fibers (POFs) [13]. The curvature of the fiber enables its power attenuation due to macrobend radiation losses [14]. The macrobend principle has been applied to temperature sensors based on POFs [1,3]. In this approach, the fiber is bent, and a slight twist is made. The increase in temperature leads to a variation in the fiber numerical aperture. Although the sensors presented in [1] and [3] were able to achieve a linearity higher than 90%, these sensors only reach 50 °C in [1] and 70 °C in [3]. Furthermore, sensor behavior when the temperature is decreased was not presented. Sensor characterization in terms of temperature decline would yield information as to how the POF material behaves in terms of a hysteresis effect.

In this paper, a POF-based temperature sensor is proposed. The sensor explores the polymer response to different temperatures. Temperature increases lead to a decrease in Young’s modulus due to the viscoelastic nature of the polymer. Therefore, if the fiber is under stress due to a constant force, such stress on the fiber will change with the temperature variation. Since the stress on the fiber causes a variation in its refractive index due to the stress-optical effect [15], there will be a variation in the POF output power with the temperature variation when it is under stress. 

In order to evaluate the Young’s modulus variation, dynamic mechanical analysis (DMA) on the fiber was performed. Furthermore, the relation of the fiber mechanical property variation with its optical response is modeled. After the validation of these effects, the POF-based temperature sensor is proposed and its temperature limits and repeatability are tested, and higher temperature limits compared to other intensity-variation-based sensors are obtained. In addition, the analytical model and DMA provide a different perspective on sensor design, which, from the authors’ best knowledge, has never been reported with respect to temperature sensors based on intensity variation. 

This paper is organized as follows. Section 2 presents the characterization of the POF mechanical properties with the temperature variation and its relation to the power attenuation on the POF response. Section 3 presents the sensor development and its results, which are presented and discussed. Final remarks and future works are discussed in Section 4.

## 2. POF Sensor Characterization

The optical fiber employed on the analysis presented in this paper is a multimode HFBR-EUS100Z POF (Broadcom Limited, Singapore) with a step-index profile. This fiber presents a core made of poly(methyl methacrylate) (PMMA) with a diameter of 980 µm. The cladding of this fiber is made of a fluorinated polymer that has a refractive index lower than that of PMMA. The cladding thickness is 20 µm. Furthermore, a polyethylene coating provides a total diameter of 2.2 mm for the POF.

The described POF had its mechanical properties characterized through DMA, and the modeling of the power variation with the temperature is presented.

### 2.1. POF Mechanical Property Analysis

The DMA comprises of the application of a predefined oscillatory load over a range of temperature and frequency. This is a widely applied method to determine the mechanical properties of a viscoelastic material such as the PMMA and polyethylene of the POF employed [16]. Since the thickness of the cladding is very small compared with the core and coating, the analysis of this paper is made only with respect to the PMMA of the core and polyethylene of the coating. The analysis of variation in the mechanical properties on the fiber is made by means of a temperature scan with a constant frequency on the DMA.

Considering that a viscoelastic material presents a storage and a loss modulus on its dynamic response, the parameters obtained on a DMA include the storage modulus (*E′*) and loss modulus (*E″*). The combination of the storage and loss moduli is the dynamic Young’s modulus (E*) of the polymer (see Equation (1)):

(1) $$E^{*}=E^{\prime }+iE^{\prime \prime }.$$

The ratio between the storage and loss moduli is the loss factor (tan(*δ*)) defined in Equation (2). This is a ratio between the dissipated energy and the storage energy per cycle of applied load.

(2) $$\tan\left(\delta \right)=\frac{E^{\prime \prime }}{E^{\prime }}.$$

The Young’s modulus is divided into the loss and storage moduli due to the duality of a viscoelastic response, which is the combination of the elastic and viscous responses of the polymer. The loss modulus represents the energy loss due to the viscous effect, whereas the storage modulus represents the energy storage due to the elastic effect. Therefore, the loss modulus may be understood as the relation between the viscous and elastic components of the polymer response. For this reason, the loss factor of an elastic material is zero (tan(*δ*) = 0).

The relation between the static ($E_{0}$) and dynamic (E*) Young’s modulus is defined by the phase shift between the input stress or strain and the viscoelastic response (*δ*). For this reason, another expression for the dynamic Young’s modulus calculation is presented in Equation (3):

(3) $$E^{*}=E_{0}\cos\left(\delta \right)+iE_{0}\sin\left(\delta \right).$$

**Figure 1:**
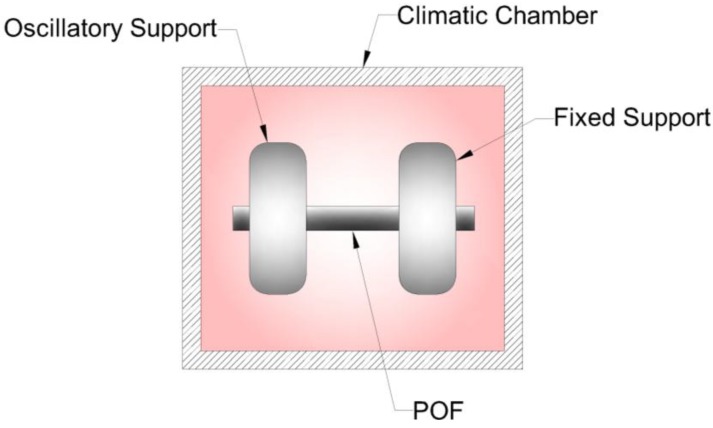
Dynamic mechanical analyzer employed on the polymer optical fiber (POF) characterization.

The dynamic mechanical analyzer employed on these tests is the DMA 8000 (Perkin Elmer, Waltham, MA, USA), which presents a temperature controller that can reach temperatures up to 400 °C with a resolution of 0.1 °C. In addition, the analyzer presents a resolution for the modulus estimation of 0.0001 Pa in a range of 10^3^–10^16^ Pa, whereas the displacement resolution is 1 nm in a range of ±1000 µm. The experimental setup is presented in Figure 1. The test consists of applying an oscillatory displacement with a constant amplitude of 0.049 mm on the POF sample. The constant frequency of oscillation is 1 Hz, and the temperature varies from 25 to 120 °C. The test was conducted following the ASTM D5418 standard employed in dynamic mechanical property analysis for polymers, where the fiber is clamped on fixed and oscillatory supports prior to the application of a predefined load of about 10 N that inhibits the sample slippage, and temperature is increased at a rate of 1 °C/min.

**Figure 2:**
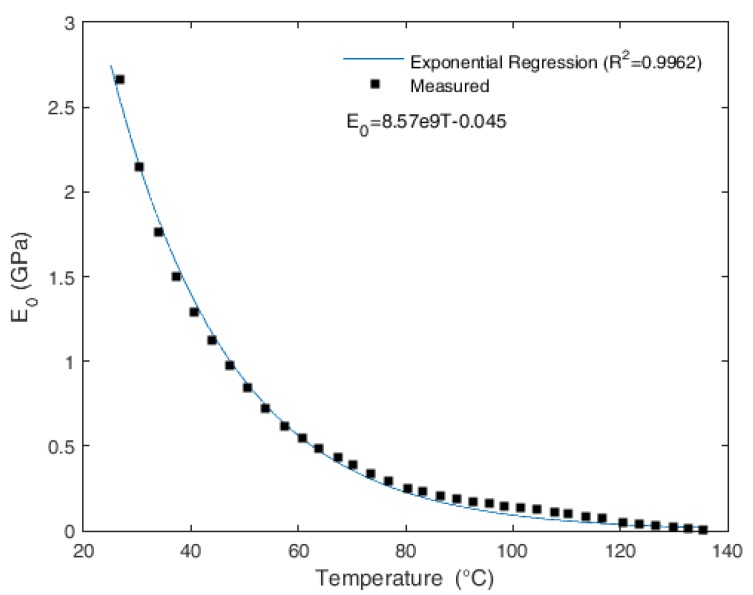
Variation of the POF static Young’s modulus with the temperature.

Figure 2 shows the variation in Young’s modulus with the temperature, which gives the static component of the Young’s modulus obtained with Equations (1) and (3).

The presented test shows a high variation in the Young’s modulus with the temperature. For instance, when the temperature of 80 °C is reached, the variation starts to decrease until a temperature around 135 °C is achieved. On this temperature, the Young’s modulus cannot be measured, since its value becomes lower than the analyzer’s resolution. Furthermore, the exponential regression between the Young’s modulus and the temperature is obtained with a correlation coefficient of 0.9962. The obtained equation is presented in Figure 2. The Young’s modulus suffers from a high temperature dependency on temperatures below 80 °C. When the temperature is close to PMMA’s glass transition temperature (about 110 °C), the variation in the Young’s modulus is lower. The melting point of the employed POF can be estimated with DMA tests at higher temperatures and is about 165 °C. In addition, a deformation of 0.05 mm was found at the end of the test, which indicates a need to position the fiber on mechanical supports so as to reduce fiber deformation and prevent errors in measurement.

### 2.2. Analysis of the POF Output Power Variation

**Figure 3:**
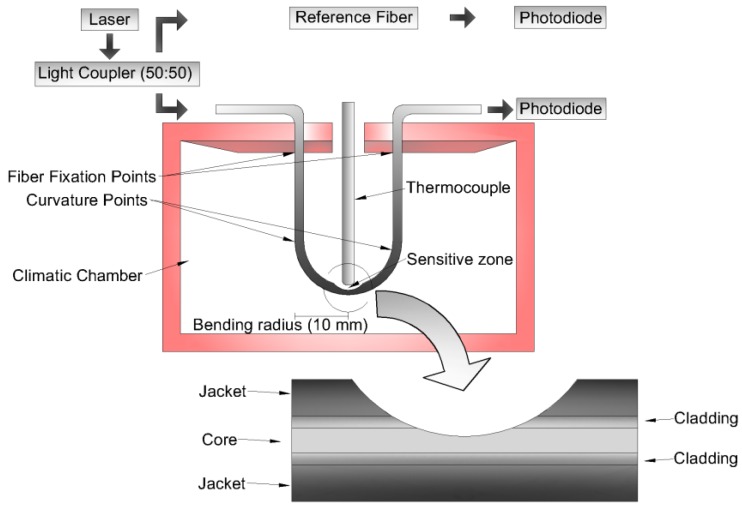
Experimental setup to evaluate the variation in the output power with the temperature sweep and the lateral view of the POF with the lateral section.

Figure 3 shows the experimental setup employed for the temperature characterization with respect to the POF output power variation and a lateral view of the fiber with a lateral section.

A laser (3 mW @650 nm) provided the light for the fiber under test and for a reference fiber, which was employed to compensate possible deviations of the light source power. The power was divided in a 50:50 coupling ratio through a light coupler IF-562 (Industrial Fiber Optics, Tempe, AZ, USA). The light power variation was acquired by two photodiodes IF-D91 (Industrial Fiber Optics, Tempe, AZ, USA), one for the reference fiber and the other for the fiber under test. The acquisition board USB-6008 (National Instruments, Austin, TX, USA) with an 8-bit analog-to-digital converter and an acquisition frequency of 200 Hz acquired the photodiode response after it was converted to voltage through a transimpedance amplifier. In order to reduce the effect of the modal distribution in the light coupler, all the optical components presented, especially the light coupler, the reference, and the sensor fibers, were carefully positioned on a support to inhibit its geometry variation. Moreover, if there was any variation in the splitting ratio of the light coupler due to modal distribution changes, a power variation was detected in the reference fiber. This power variation could be further compensated by normalizing the sensor response with respect to the reference fiber.

The fiber was bent on a 180° angle and positioned inside a climatic chamber 400/1ND (Ethik Technology, Vargem Grande Paulista, SP, Brazil), which had a closed loop control with a resolution of 0.1 °C and could reach a maximum temperature of 200 °C. A thermocouple was positioned close to the fiber curvature region to provide the reference temperature for analysis, which presented a temperature resolution of 0.1 °C and a temperature range of 220 °C. A lateral section was made on the fiber to create a sensitive zone, which could increase sensor sensitivity, since the stress on the fiber depends on the cross-sectional area [17]. The increase in the stress on the fiber, by means of the additional curvature applied and by the lateral section made, could increase the stress-optical effect, which led to a higher variation in output power.

The curvature applied to the POF created a stress on the fiber. If pure bending stress was considered, the static stress (*σ*_0_) was calculated as Equation (4):

(4) $$\sigma_{0}=\frac{E_{0}x}{R}$$

where *R* is the distance between the fixation points and the sensitive zone (60 mm), and *x* is the perpendicular distance between the bending part and the POF center, which is about 180 mm in the case presented in Figure 2. Equation (4) shows that a variation in the Young’s modulus leads to a variation in the stress on the fiber.

The relation between the stress (*σ*_0_) and the refractive index variation (∆*n*) is presented in Equation (5), where *q*_11_ is the stress-optical coefficient, which is about 10–11 Pa-1 for polymers [15], and *n_c_* is the core refractive index, which is 1.49 for this optical fiber.

(5) $$∆n=\frac{n_{C}^{3}q_{11}\sigma_{0}}{2}.$$

The effective refractive index is the core refractive index subtracted by the refractive index variation. This effective refractive index (*n_eff_*) is substituted in the Snell law to obtain the critical angle (see Equation (6)) and in the equation of the bending correction for the critical angle presented in Equation (7) [18]:

(6) $$\theta_{c}=\cos^{‒1}\left(\frac{n_{cl}}{n_{eff}}\right)$$

(7) $$\theta_{b}=\theta_{c}\sqrt{1‒\frac{2a}{R\theta_{c}^{2}}}.$$

In Equations (6) and (7), *a* is the core radius (0.49 mm), and *n_cl_* is the cladding refractive index (1.417).

Finally, Equations (6) and (7) are substituted in Equation (8) to obtain the POF curvature sensor output power (*P_o_*) [18]:

(8) $$\frac{P_{o}}{P_{i}}=\frac{\left(S_{C}‒S_{o}\right)\sin^{2}\left(\theta_{b}\right)}{S_{C}\sin^{2}\left(\theta_{c}\right)}$$

where *S_C_* is the cross-sectional area of the core, *S_o_* is the cross-sectional area of removed material on the sensitive zone, and *P_i_* is the input power from the laser.

**Figure 4:**
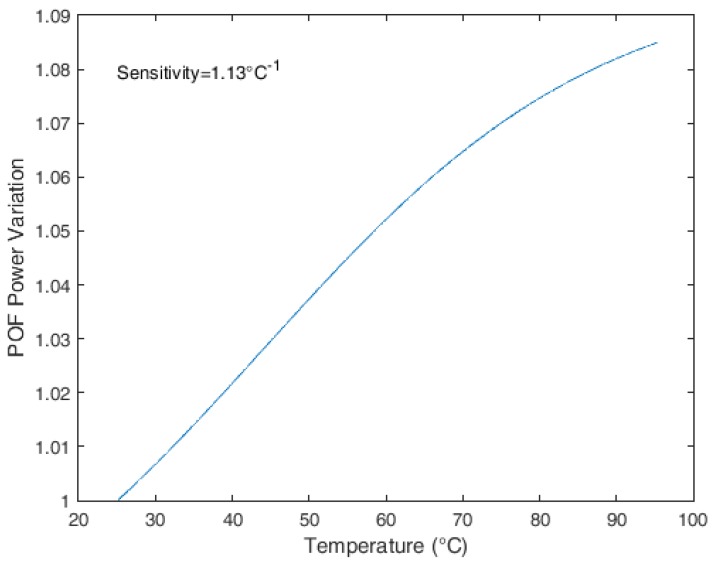
An analytical model of POF power variation under curvature with the temperature increase.

The presented model is applied with the variation in the Young’s modulus with the temperature available on the DMA tests and a sensitivity of 1.13 °C^−1^ is obtained. Figure 4 shows the results for the temperature variation from 25 to 97 °C. The Young’s modulus applied to the model is the one obtained in DMA experiments (see Figure 2), and the lower bound of the result (25 °C) is the room temperature (rt) of the characterization on the analyzer. The limit of 97 °C is the one where the Young’s modulus is close to the analyzer resolution and is close to the PMMA glass transition temperature (T_g_). Since the Young’s modulus decreases with the increase in temperature, the stress on the fiber decreases, which leads to a reduction in the attenuation of the power when compared with the fiber in higher temperatures.

## 3. POF Temperature Sensor Tests

The application of a constant stress on the fiber increases its sensitivity to temperature variations. However, increasing temperature sensitivity by submitting the fiber to a higher stress can cause irreversible changes in the POF material. For a curvature sensor under stress with a temperature higher than rt, if the temperature decreases to rt, the POF response may not decrease to its original value. Therefore, in order to obtain a temperature sensor based on POF material features, it is necessary to apply stress to the fiber and position the fiber such that it does not change its curvature when its stiffness is reduced due to the temperature increase. Thus, the sensor will have less sensitivity with respect to the temperature variation when compared to the sensitivity that such a sensor might have if the maximum stress that the POF tolerates is applied. However, it will increase the sensor repeatability and enable the sensor to measure heating and cooling processes. For this reason, the curvature radius applied is higher than the ones presented in [1,3].

**Figure 5:**
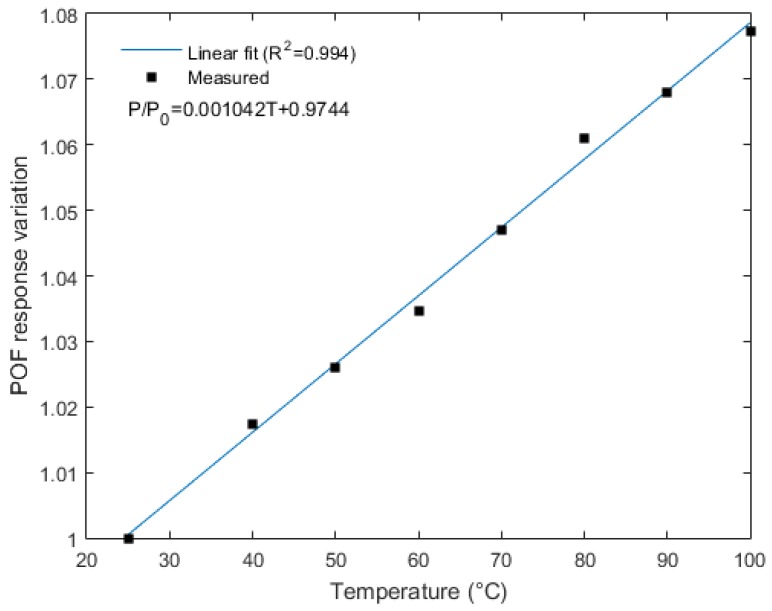
Calibration curve for the POF temperature sensor.

The temperature sensor developed has the same setup presented in Figure 3. The first test on the sensor saw a temperature increase from rt (about 25 °C) to 100 °C. The rt and the temperatures of 40 °C, 50 °C, 60 °C, 70 °C, 80 °C, 90 °C, and 100 °C were acquired by the temperature sensor of the climatic chamber and a thermocouple positioned close to the sensor. The POF sensor response was compared with the temperature measured by the thermocouples and a linear regression was made (see Figure 5).

The sensor presented a sensitivity of 1.04 × 10^−3^ °C^−1^ and a linearity of 0.994. In addition, the root mean squared error (RMSE) of the proposed sensor was 1.48 °C. Comparing with similar sensors presented in the literature, the sensitivity was higher than the one presented in [3] (8.95 × 10^−4^ °C^−1^) and was close to the one presented in [1], which was 1.29 × 10^−3^ °C^−1^ for a curvature radius of 2 mm. The curvature radius of the sensor presented in this work was 10 mm. Furthermore, the sensor presented had higher linearity than the one discussed in [1] (0.961) and similar linearity when compared with the sensor presented in [3] (0.995). The higher linearity of the sensor when compared to the characterization tests is related to the lower accommodation of the fiber due to the stress applied and the influence of the shear stress, which was caused by the fiber bending on the sensor response.

The sensor repeatability was evaluated in five tests. Each test consisted of increasing the temperature to a predefined value and then decreasing it to the initial value. These tests were conducted to evaluate the capacity of the sensor to measure the heating and cooling processes and to set the operation limits of the sensor. The chosen temperature cycles were about 40 °C, 65 °C, 80 °C, 110 °C, and 140 °C. Although it was not assessed in POF response characterization or in the sensor calibration tests, a temperature of 140 °C was tested to evaluate the operation limit of the sensor.

**Figure 6:**
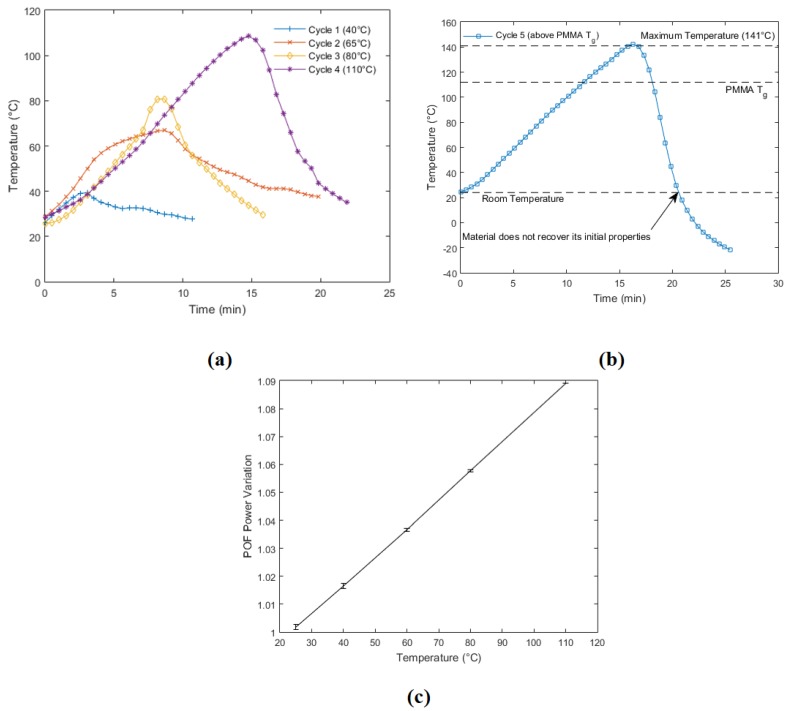
Repeatability tests for the POF temperature sensor. (**a**) Cycles ranging from 40 to 110 °C to show the sensor behavior with increasing and decreasing temperatures. (**b**) Sensor response on a test with temperatures higher than the PMMA T_g_. (**c**) Repeatability of the temperature sensor on the cycles presented in (**a**).

Figure 6 shows the five cycles performed on the sensor. The temperature limits of these cycles were due to environmental conditions and the operational limitations of the heater employed. Cycle 1 was made between 26 and 39 °C. Cycle 2 started at 28 °C, and its maximum temperature was 66 °C. Cycle 3 started at the same temperature as Cycle 1, and its maximum temperature was 80 °C. Cycle 4 started at 28 °C, the temperature was increased to about 110 °C, and, after achieving its maximum value, the temperature was decreased back to the initial value. Finally, Cycle 5 started at 24 °C and achieved 141 °C. Figure 6a shows the responses of Cycles 1–4, which are the ones below the PMMA glass transition temperature and presented similar behavior with the temperature variation. Figure 6b presents the result of Cycle 5, where a temperature higher than the polymer T_g_ was applied, and it can be seen that the sensor was not able to recover to its initial temperature (25 °C). This behavior is represented by an offset due to variations in the polymer properties when it was submitted to temperatures higher than T_g_. In addition, this offset may also have occurred due to another phase transition that can occur at high temperatures, where there is a stretching of the polymer chains, which leads to irreversible changes of the sensor response. Finally, Figure 6c shows the response of the first four cycles with respect to the temperature, where it is possible to evaluate sensor repeatability. The error bars represents the deviation of the sensor response at each cycle. Although the maximum deviation among the temperature tested occurred at 40 °C, such variation represents only about 1% when the four cycles are compared.

The only cycle that the temperature not returned to its original value was Cycle 5. This might have been due to the glass transition of the polymer, which led to an irreversible variation in its properties. Since 141 °C is higher than the POF glass transition temperature [19], the sensor did not recover its initial mechanical properties and led to errors in measurement. For this reason, the sensor operation may be limited to 110 °C, which is higher than the one obtained in the literature for similar sensors [1,3]. Although the tests were conducted at temperatures higher than rt, lower temperatures can also be measured, which would lead to a reduction in POF power proportional to the temperature. In addition, negative temperatures as low as −55 °C can also be measured [3]. 

The response of each cycle shows repeatable behavior of the sensor, especially in the heating process. The only response that shows a slight deviation of this behavior is Cycle 2. Since the response of each sensor is shown with respect to time, the deviations in Cycle 2 are related to deviations in the heating process of the heater employed. Furthermore, the employed heater does not have a controllable cooling system. For this reason, the cooling process is limited to the natural convection between the heater and the room, which not only increase the time of cooling but also make this process less repeatable. Nevertheless, the results presented in Figure 6c show a high repeatability of the sensor with deviations of about 1% between the four cycles made.

## 4. Conclusions

This paper presents the characterization and development of a POF temperature sensor. The sensor is based on variation in the POF mechanical properties with temperature variation. If the fiber is under a constant stress, the variation in its mechanical properties leads to a variation in output power due to the stress-optical effect. The POF mechanical property variation is characterized in a DMA. An analytical model for the sensor behavior is proposed and simulated. Such a model is validated in temperature tests with the proposed sensor.

Tests to obtain the sensor calibration curve and to evaluate its operation limits were conducted, and the sensor presented a sensitivity and linearity higher than those presented in the literature. Furthermore, the sensor is able to measure temperature until 110 °C, which is higher than the limit obtained in similar sensors presented in the literature. This temperature limit is due to the PMMA glass transition temperature and by applying POFs with materials that present higher glass transition temperatures, such as the step-index Topas/Zeonex [20] and CYTOP [21] fibers, the temperature limit of the proposed sensor can be further increased. Nevertheless, the sensor proposed can operate at the same range of temperatures of the PMMA polymer optical fiber Bragg grating (POFBG) with similar linearity [22], but with the additional advantage of a cost that is some orders of magnitude lower.

Future works include improvements on the hardware for the signal acquisition, which can enable the improvement of the sensor resolution. Furthermore, a heater with cooling capabilities and higher temperature precision will be applied to further evaluate the sensor with respect to its resolution and hysteresis.
